# Bisphosphonate-induced Atypical Femoral Shaft Fracture

**DOI:** 10.7759/cureus.1750

**Published:** 2017-10-05

**Authors:** Muhammad Usman Baig, Awais Maqbool

**Affiliations:** 1 Internal Medicine, Bahawal Victoria Hospital, Quaid-E-Azam Medical College, Bahawalpur, PAK; 2 Multan Medical College, Multan, PAK

**Keywords:** bisphosphonates

## Abstract

Bisphosphonates are used to treat postmenopausal and glucocorticoid-induced osteoporosis, Paget's disease of bone, and malignant hypercalcemia. They have been related to atypical femur fractures. We describe an 83-year-old woman on long-term bisphosphonates presenting with an atypical femur fracture. She underwent a surgical procedure and, a few months later, she presented with a nonunion of the periprosthetic femoral fracture. She underwent an additional surgical procedure and discontinued bisphosphonate treatment. As more atypical femur fractures associated with bisphosphonate use are being reported in the literature, the risk of an atypical femur fracture with bisphosphonate use should not be ignored.

## Introduction

Osteoporosis is an important health challenge, and bisphosphonates provide the most benefit at the least cost. Structurally, bisphosphonates are analogues of pyrophosphate, with the feature of being resistant to enzyme or chemical breakdown. The bisphosphonates act by inhibiting the resorption of bone by osteoclasts; as a result, inhibiting bone mineralization and decreasing bone turnover.

The medical literature reports that bisphosphonates are a potential cause of atypical femur fractures. Atypical fractures are characterized by transverse and noncomminuted fracture patterns in the subtrochanteric or femoral shaft regions with a medial cortical spike at the fracture area. Other features include prodromal pain and a generalized thickening of the femoral cortices on radiographs [1-2]. We observed most of these features in the present case.

## Case presentation

**Figure 1:**
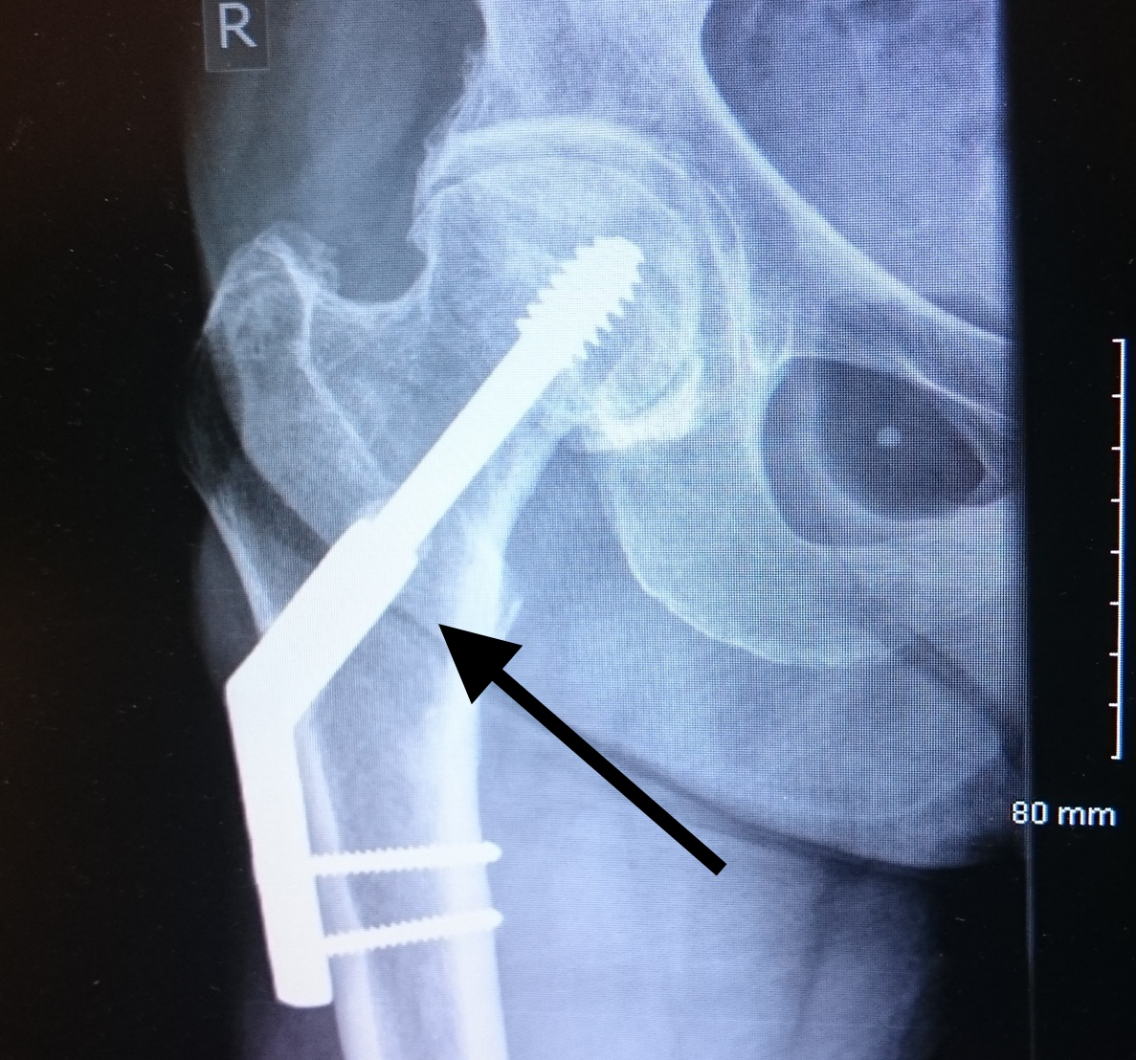
Dynamic hip screw with a two-hole plate for the neck of the femur fracture Arrow showing the two-hole dynamic hip screw and plate

**Figure 2:**
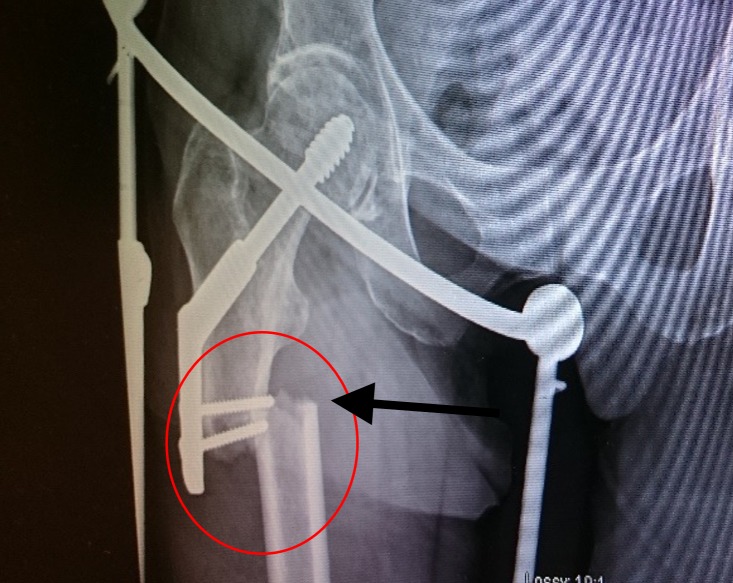
Subtrochanteric femur fracture Red circle and arrow denoting the subtrochanteric fracture

An 83-year-old woman presented to our hospital with low-energy trauma resulting in pain in the right thigh, causing an inability to bear weight. She has a history of chronic obstructive pulmonary disease, type 2 diabetes mellitus, gastroesophageal reflux disease, and osteoporosis. She had an intertrochanteric fracture in the right neck of her femur in 2010, which was internally fixed using a dynamic hip screw (DHS) with a two-hole plate (Figure 1, Figure 2).

She was started on alendronate therapy in 2011 for osteoporosis. She had a fracture in the left distal radius in 2013 and underwent manipulation under anesthesia and Kirschner wire fixation. She experienced low-energy trauma in January 2015 and presented with right thigh pain and inability to bear weight.

**Figure 3:**
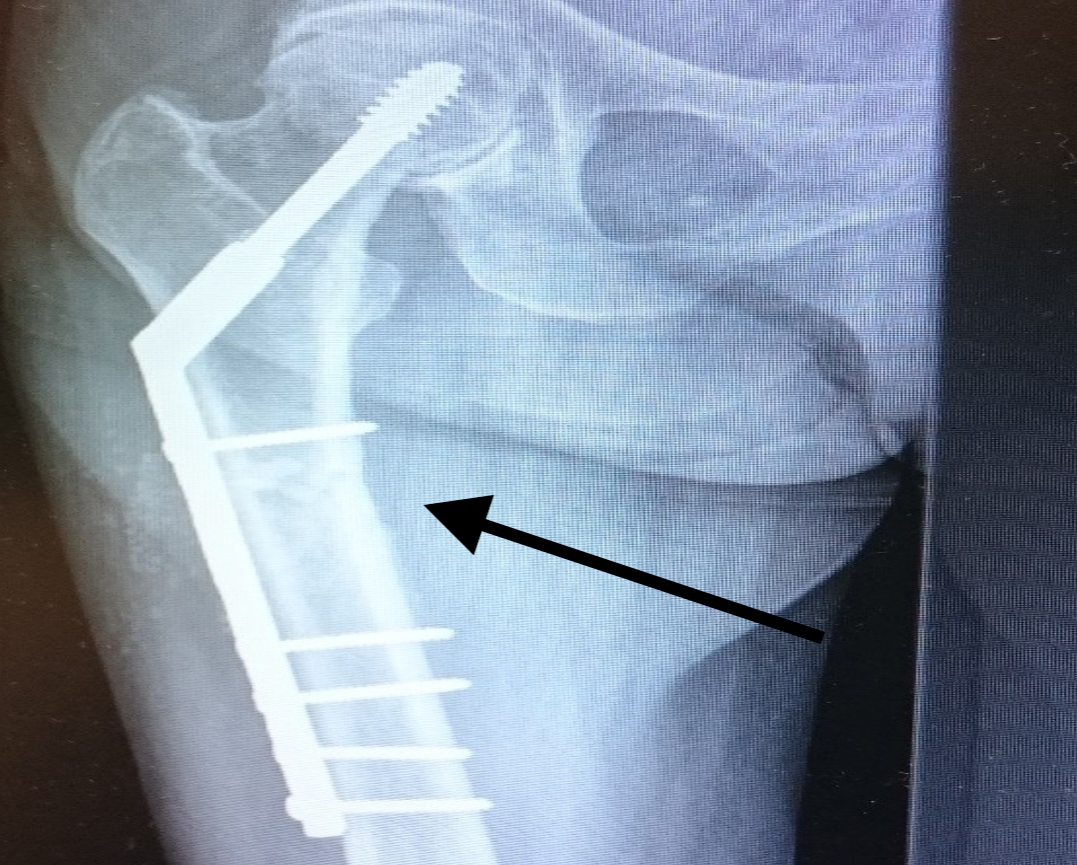
Conversion to the dynamic hip screw with a 10-hole plate Arrow showing fracture fixation with a 10-hole dynamic hip screw (DHS) plate

**Figure 4:**
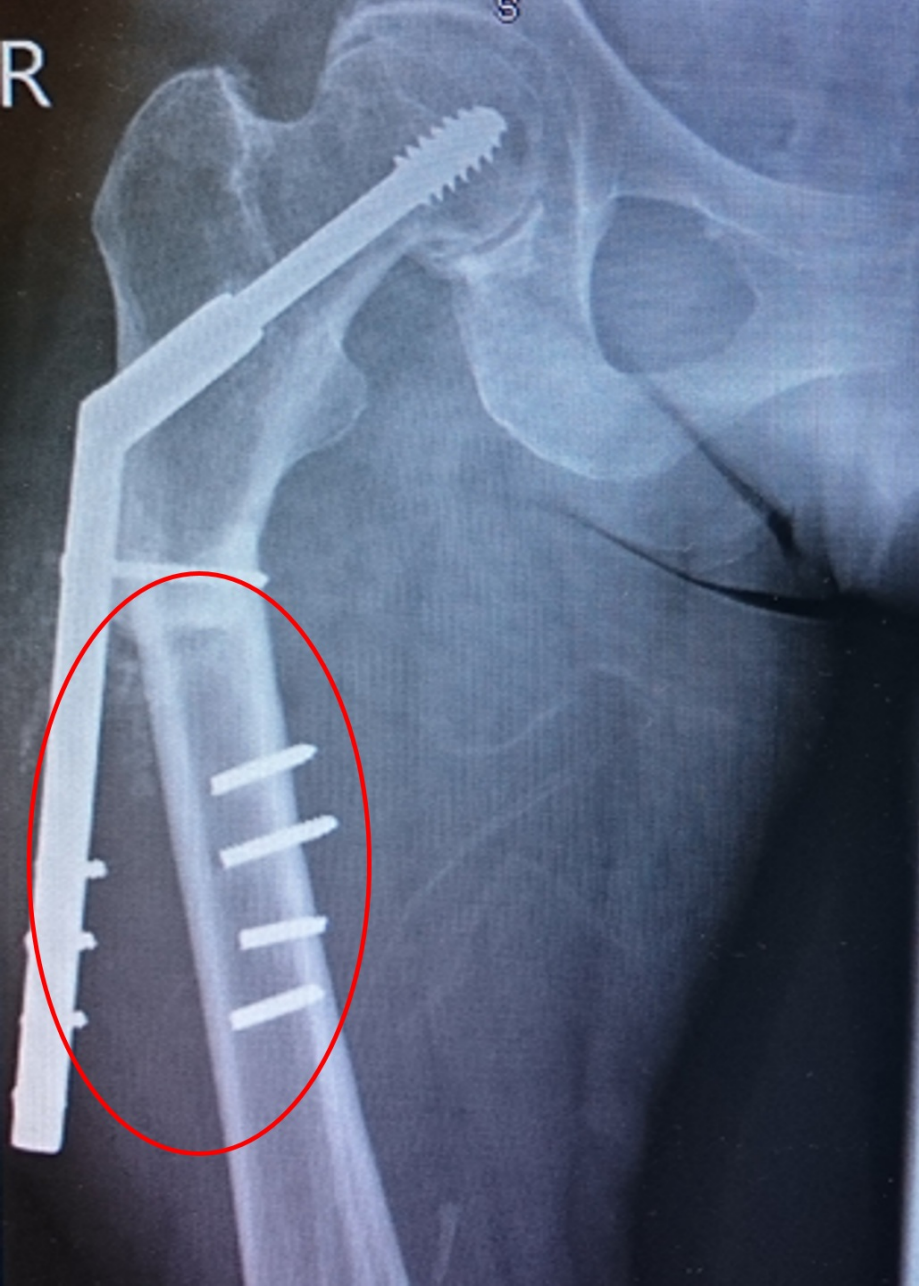
Periprosthetic fracture Red circle denoting implant failure

Radiological evaluation of the right femur and pelvis showed an atypical subtrochanteric femur fracture of the right leg (Figure 3). She underwent a surgical operation the next day, and a two-hole plate was removed without removing the lag screw and replaced using a DHS with an eight-hole plate (Figure 4). She started physiotherapy, and within few weeks, she was walking with a walking frame. She received regular follow-up radiological examinations.

**Figure 5:**
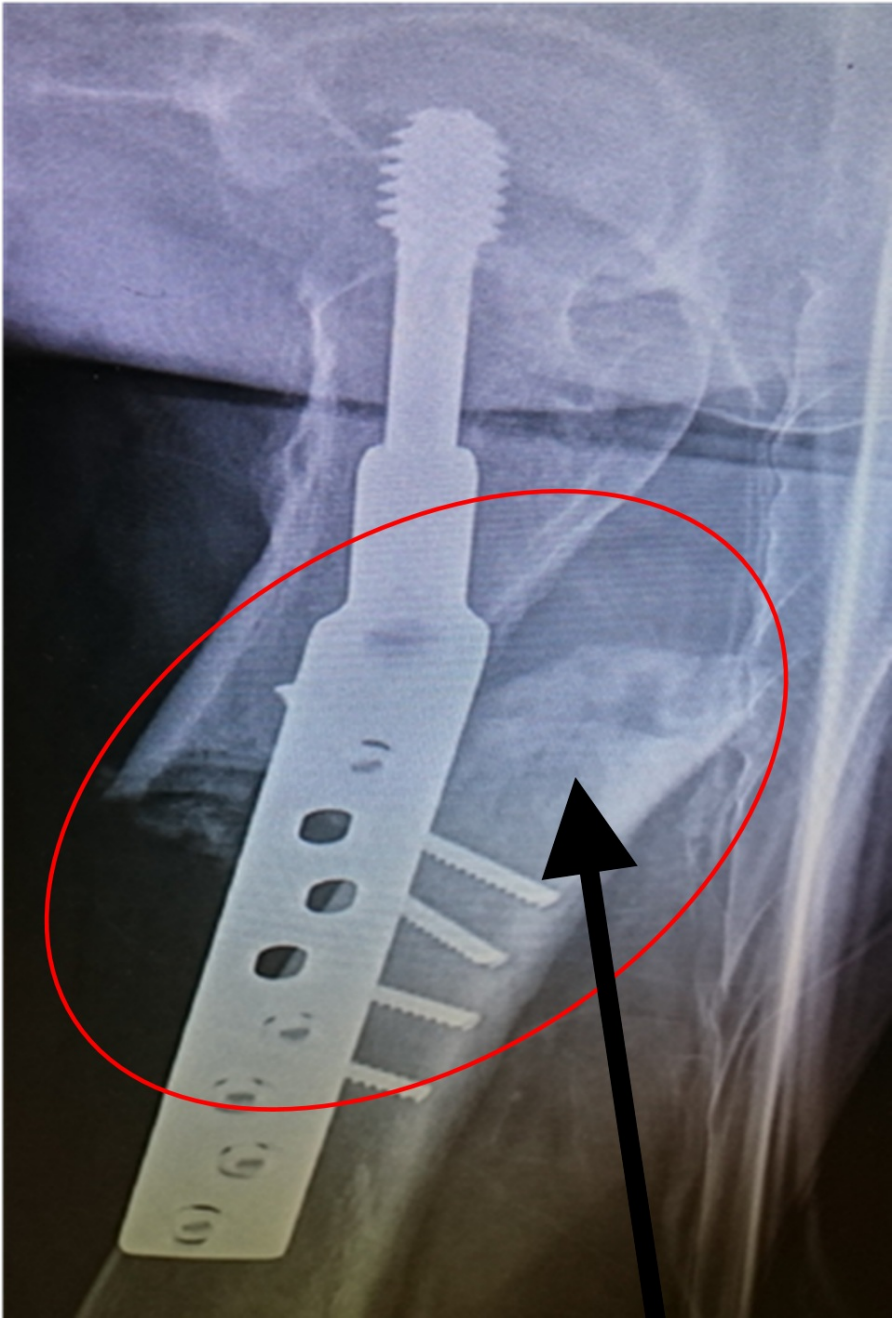
Lateral view of the periprosthetic fracture Red circle and arrow showing the displaced periprosthetic fracture

**Figure 6:**
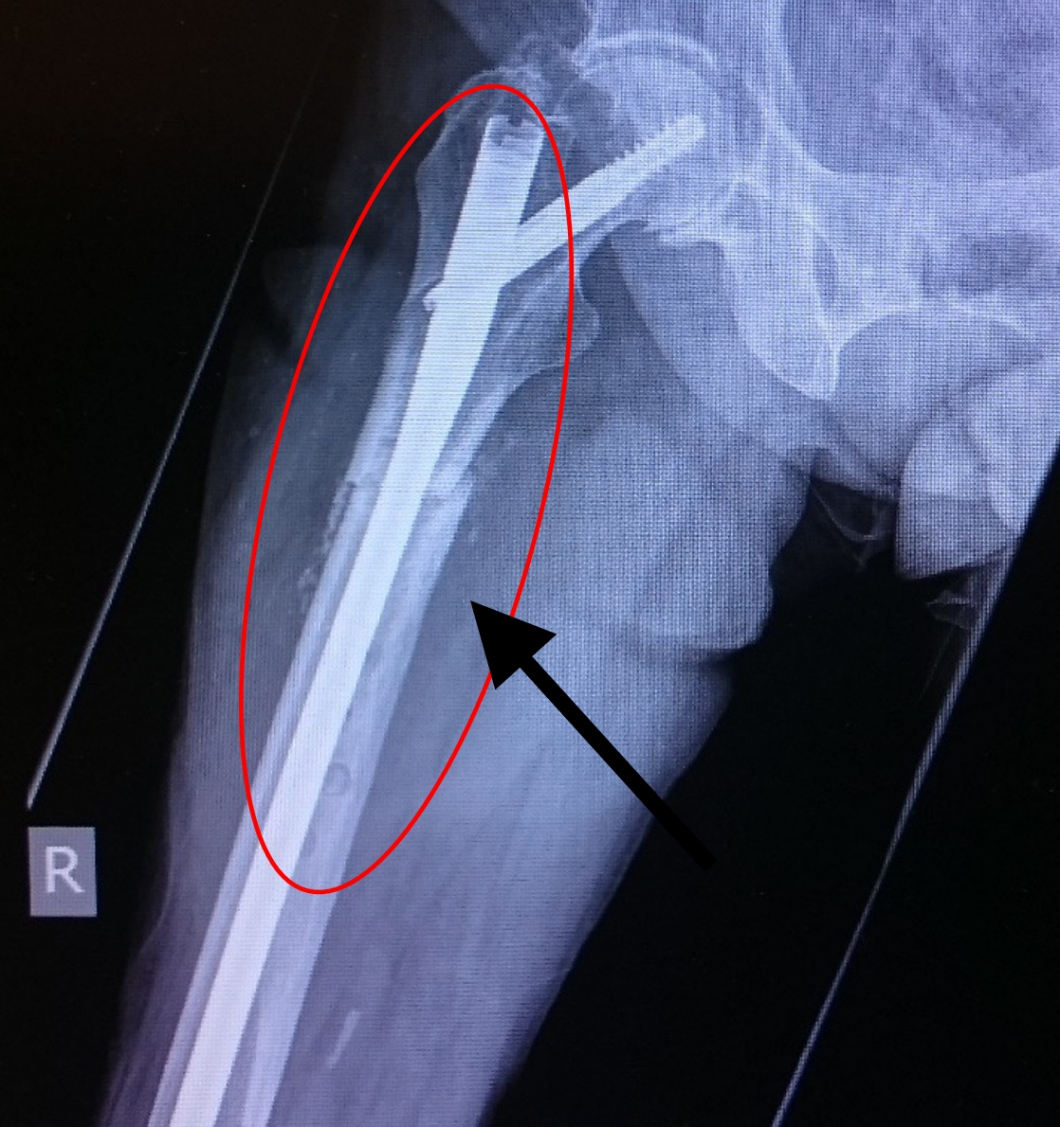
Cephalomedullary nailing of the fracture Red circle and arrow showing the intra-medullary nailing

Her alendronate was discontinued, and she was started on teriparatide injections. Teriparatide is a synthetic parathyroid hormone that exists naturally in the body. She was undergoing this treatment when, in April 2015, she had another low-energy fall and was brought to the hospital with pain in the right thigh and an inability to bear weight. On radiologic examination, she had DHS implant failure and nonunion of the subtrochanteric fracture (Figure 5 ). She underwent an additional surgical procedure, and the DHS was removed. A long cephalomedullary nail was inserted (Figure 6 ). She started physiotherapy again. The patient is receiving regular outpatient follow-up and radiologic examinations. She is walking with a walking frame and continues to receive teriparatide injections.

## Discussion

Bisphosphonates are the primary pharmacological agents used to treat osteoclast-mediated bone loss due to osteoporosis, Paget’s disease of bone, malignancies that metastasize to bone, multiple myeloma, and hypercalcemia of malignancy. Apart from the aforementioned uses, it is also used for low bone density and osteogenesis imperfecta as well [3-4].

Since their introduction into clinical practice, bisphosphonates have transformed the clinical care of an array of skeletal disorders characterized by excessive osteoclast-mediated bone resorption. Bisphosphates act as strong inhibitors of bone resorption by suppressing the activity of osteoclasts, and although this improves osteoporotic conditions, it reduces overall bone turnover [5]. Prolonged bisphosphonate-induced impairment of bone remodeling may cause an accumulation of micro-fractures and weakening of bone [6-7].

Atypical femur fractures are associated with the duration of bisphosphonate therapy [8]. The risk of atypical femur fracture had previously been associated with the use of glucocorticoids and proton-pump inhibitors [8]. It has been suggested in the literature that the use of bisphosphonates should be evaluated after a certain period of time [6]. Bisphosphonates are very effective for the first few years in patients with osteoporosis by preventing osteoporotic fragility fractures [9-10]. Consideration should be given to stopping bisphosphonate therapy, at least temporarily, in those patients who are assessed to be at low or low-to-moderate risk, defined as: (1) no incident fractures, (2) T-score > −2.0, and (3) no other major risk factors after a 3- to 5-year therapeutic period [8].

## Conclusions

The use of bisphosphonates is beneficial for osteoporosis, but after a certain period, it can increase the risk of atypical femoral shaft fractures. Therefore, it is important for both clinicians and patients to use bisphosphonates for the correct indications but to bear in mind the duration of its use as well.
